# Use of contrast-enhanced ultrasound for assessment of nodular lymphoid hyperplasia (NLH) in canine spleen

**DOI:** 10.1186/s12917-019-1942-5

**Published:** 2019-06-11

**Authors:** Cyndi Mangano, Francesco Macrì, Simona Di Pietro, Michela Pugliese, Silvia Santoro, Nicola M. Iannelli, Giuseppe Mazzullo, Rosalia Crupi, Massimo De Majo

**Affiliations:** 1 Department of Veterinary Sciences, University of Messina, Polo Universitario Annunziata, 98168 Messina, Italy; 2 Camagna Veterinary Clinic, Via Parco Caserta 13, RC 89124 Reggio Calabria, Italy; 3 Department of Chemical, Biological, Pharmaceutical and Environmental Sciences, University of Messina, Viale F. Stagno d’Alcontres 31, 98168 Messina, Italy

**Keywords:** Dog, Contrast-enhanced ultrasonography, Diagnostic ultrasound, Nodular lymphoid hyperplasia, Spleen, Sonovue, Qontrast

## Abstract

**Background:**

Nodular lymphoid hyperplasia (NLH) is one of the most common non-neoplastic splenic lesions in dogs, especially in old ones, showing a splenic enlargement. More recent studies have been focused on Contrast Enhanced Ultrasonography (CEUS) analysis of the spleen for establishing normal perfusion patterns and blood pool phase peculiarities of focal lesions.

The aim of the study was to evaluate the qualitative and quantitative CEUS analysis of the canine splenic NLH, characterizing the CEUS pattern of this pathology on 20 clinical cases.

**Results:**

A prospective, observational study was performed using a system equipped with contrast-tuned imaging technology. Mechanical Index was set from 0.08 to 0.11; the contrast medium was a second generation contrast medium composed of sulphur hexafluoride encapsulated of a shell of phospholipids (SonoVue®). Qualitative and quantitative assessment of the enhancement pattern of splenic NLH were performed.

Cytology and histology identified 20 splenic NLH. All of the benign hyperplastic lesions assessed were isoechoic with a homogeneous pattern than the surrounding normal spleen, during the wash-in phase (10–20 s) of the CEUS exam. Before finishing the wash-in phase, 20–45 s from the contrast medium inoculation, 19/20 benign nodules became markedly hypoechoic to the adjacent spleen. Sensitivity of hypoechoic pattern for NLH was 95%.

**Conclusions:**

These findings should prove useful in the evaluation of focal splenic masses in dogs. Since enhancement and perfusion patterns of NLH seem to coincide with some neoplastic lesions of the spleen previously reported, in clinical practice attention must be paid to the final diagnosis of canine splenic lesions using only the CEUS exam.

## Background

Morphological changes of the splenic tissue related to the presence of focal lesions are common in aging dog [1].

Nodular lymphoid hyperplasia (NLH) is one of the most common non-neoplastic focal splenic masses, especially in old animals showing enlarged spleen [2–4].

Ultrasonography is the imaging technique of choice for the detection of splenic disease, even though it has a low specificity.

Nodular hyperplasia has a variable appearance and cannot be differentiated on the basis of ultrasonography alone. Echogenicity of the nodules may be similar to the remainder of the spleen; irregular with a hyperechoic central region surrounded by a thin hypoechoic rim; well-demarcated with centralized hypoechoic areas; hyperechoic [5]. Sometimes the nodules are not immediately detected in the normal parenchyma and their presence can be suspected by a solid nodule bulging from a regular border of the splenic capsule [1].

Application of Doppler ultrasound allowed to evaluate the characteristics of large vasculature network within and adjacent to the focal splenic lesions [6] .

At Computed Tomography, malignant splenic masses had significantly lower attenuation values than nonmalignant splenic masses, on both pre- and postcontrast images [7], although a recent study reported substantial overlap in the pre- and postcontrast CT features of malignant and nonmalignant splenic masses and provides limited specific diagnostic information [8].

The development of microbubbles contrast media and the use of harmonic technique allowed to assess microcirculation. Moreover, by enhancement kinetics, contrast ultrasound provided a mean of quantification of tissues perfusion [9].

More recent studies have been focused on Contrast Enhanced Ultrasonography (CEUS) analysis of the spleen for establishing normal perfusion patterns and blood pool phase peculiarities of focal lesions, with the purpose of overcoming the limitations of the standard ultrasonography altogether and replace invasive or very expensive procedures in discriminating between malignant versus benign splenic lesions [10–15].

Several enhancement patterns have been observed in the benign focal lesions: isoechogenicity with the surrounding normal spleen in most of them; hypoechogenicity (marked enhancement of lesion with marginal hypoechogenicity) throughout all blood pool phases and hyperechogenicity in the early arterial phase have also been detected in a few of them [13, 14].

Morphological or functional modifications of the vascular network have been supposed to be responsible of the enhancement pattern variations in splenic lesions [12]. Alterations of lymphoid tissue have been suggested to vary vascular bed and blood flow dynamics of the spleen. Indeed, both the decrease of the venous sinusoids and the formation of hematic lacunae have been found concurrent with lymphoid hyperplastic foci [10, 12, 16–20].

Based on the aforementioned concerns, NLH enhancement and perfusion patterns during CEUS analysis thus far appear to be neither specific nor characterized in depth and deserve more attention in order to avoid misdiagnosis [12–14].

To testing a previously developed diagnostic criteria, we performed a CEUS examination in 20 dogs affected by NLH, we also reported the quantitative CEUS in order to provide the quantification of blood flow parameters of this lesion.

## Results

All dogs included in the study were considered healthy and their haematological and haematochemical parameters were within reference range.

On B-mode examination, dogs enrolled had a single focal lesion with a mean diameter of 1,86 cm (range: 0.45–3.04 cm). 5/20 nodules were located near the splenic capsule, giving an alterated splenic border. 10/20 nodules were homogeneous and hypoechoic than the splenic parenchyma, 8/20 nodules had mixed echogenicity, only 2 lesions were isoechoic with small hypoechoic areas.

Color and Power Doppler showed detectable vessels only in 10/20 lesions, with small vessels entering the lesion and in 3/10 with vessels followed the contour of the lesion.

Contrast-enhanced ultrasound of the normal splenic tissue showed rapid enhancement of the small splenic arteries (10–13 s), a heterogeneous phase of enhancement of the spleen that became homogeneous at peak enhancement and a slow decrease of enhancement.

During the wash-in phase (10–20 s) of CEUS exam the benign hyperplastic lesions (20/20) were isoechoic with a homogeneous pattern than the surrounding normal spleen.

**Fig. 1:**
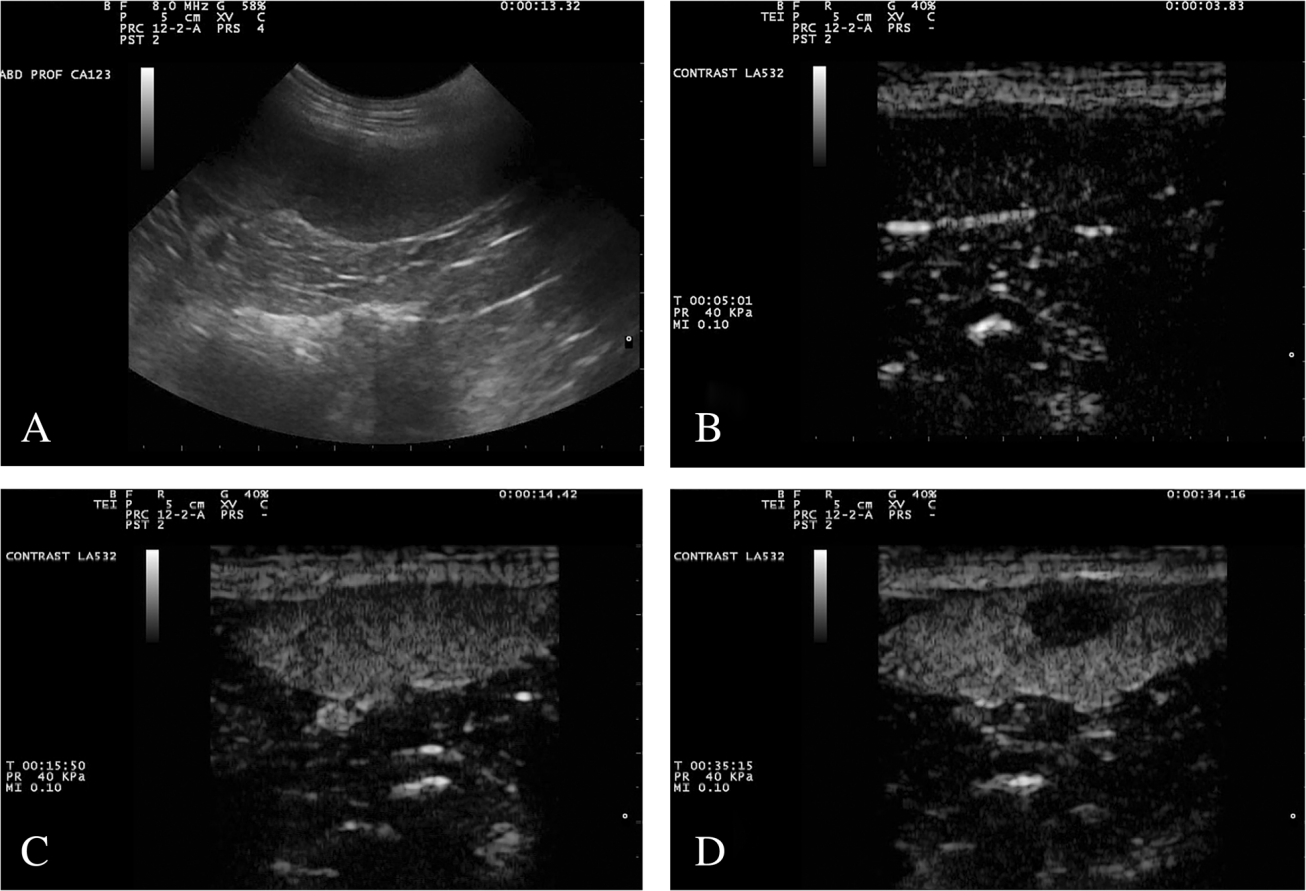
Gray-scale (**a**) and contrast enhanced ultrasound (**b**–**d**) images of a splenic benign nodular hyperplasia. The lesion is a round hypoechoic nodule in fundamental ultrasound (**a**). In the first seconds of the contrast-enhanced ultrasound examination (**b**), before the entrance of the contrast medium, the image is black because of the suppression of the fundamental signal. After about 10 s (**c**), the nodule is isoechoic to the surrounding spleen, but it becomes completely hypoechoic after a few more seconds (**d**)

Starting 20–45 s from the contrast medium inoculation, when enhancement of the normal spleen was becoming homogeneous until peak enhancement (about 40 s after injection), 19/20 (95%) benign nodules became markedly hypoechoic to the adjacent spleen. Inner thin vessels enhancement were visible at this time, even though they gradually disappeared during the wash-out phase of the normal spleen, so that lesions acquired anechoic feature (Fig. 1).

Only 1 nodule was isoechoic during all blood pool phases of the CEUS exam.

Sensitivity of hypoechoic pattern for benign nodular hyperplasia was 95% (95% CI 86–100).

**Fig. 2:**
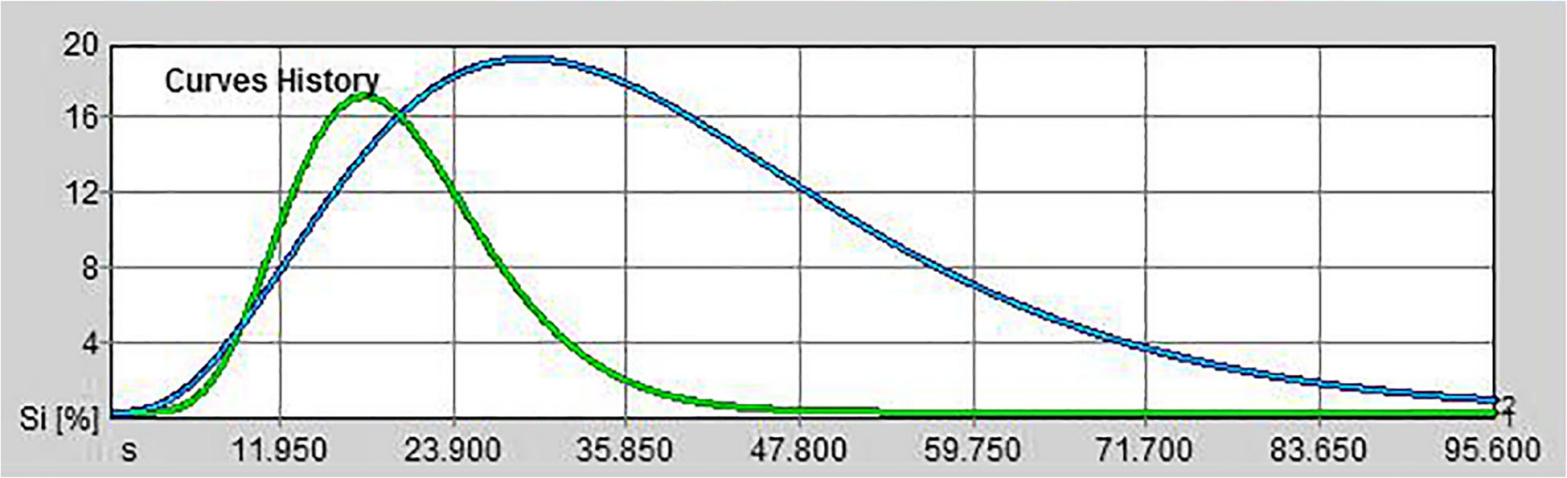
Quantitative analyses performed after injection of contrast agent with dedicated perfusion software. Time (seconds) vs. signal intensity (SI%, vertical axis) parametric graphs were drawn from ROIs positioned in the splenic nodule (green graph) and in the normal spleen parenchyma (blue graph)

Post-processing quantitative analysis showed, a TIC characterized by a faster wash-in and an early washout than normal parenchyma (Fig. 2).

The ascending segment of the curve was steep and then the curve rapidly descended to baseline.

**Fig. 3:**
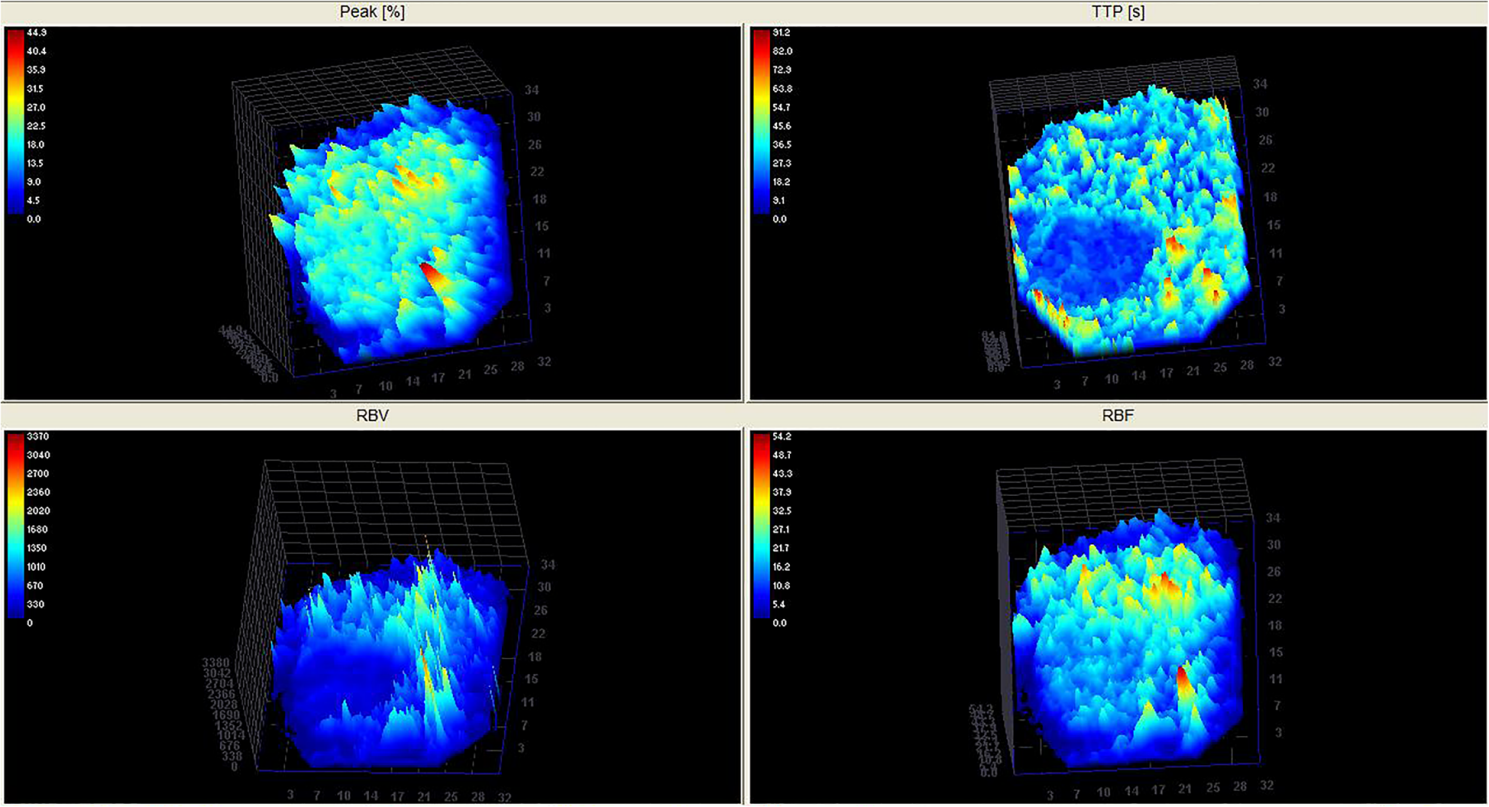
Three-dimensional CEUS reconstruction (colour-coded perfusion map) demonstrating Peak (%), TTP(s), RBV and RBF of a canine spleen with a hyperplastic nodule expressed in arbitrary units of signal intensity

The three-dimensional images of Time to Peak provided a more intuitive visual representation of the contrast medium flow patterns, along the time, both in the nodular tissue and the surrounding normal spleen (Fig. 3).

Descriptive statistical analysis of the quantitative CEUS-derived parameters revealed a normal distribution for each value. The perfusion values were significantly lower in the nodule tissue compared with the values of the normal spleen. The values of median, range and P statistical significance of perfusion parameters for both lesion and normal splenic tissues are provided in Table 1.

**Table 1:** Median, range values and *P*-value of CEUS perfusion parameters in Nodular Lymphoid Hyperplasia (NLH) and surrounding normal parenchyma

Variables	NLH lesion	Normal parenchyma	*P*-value
PI (%)	16.2 (10.1–23.7)	20.15 (14.9–31.6)	0.0001
TTP (s)	27.13 (10.54–65.49)	49.53 (25.76–59.38)	0.03
MTT (s)	37.13 (16.47–88.51)	59.72 (34.33–101.32)	0.004
RBF	15.89 (10.88–28.67)	24.72 (16.84–40.52)	0.0001
RBV	669.65 (196.44–2256.1)	1767.6 (616.83–4109.36)	0.0001

All the examined smears showed cytological features common in all the investigated cases.

They showed a variably degree of cellularity but even optimal quality and moderate hemodilution.

All smears were characterized by a mixed lymphoid population mainly represented by a predominance of medium and small lymphocytes, a certain presence of lymphoblasts, rare plasma cells or other kind of cells. No signs of malignancy were observed.

These findings recalled the features of a typical hyperplastic lymph node response; thus, on this basis and on mere cytological observation, they strongly suggested a diagnosis of hyperplastic nodular reaction of the spleen.

None of dog had adverse reaction during the procedure. B-mode follow up examination after one month showed unremarkable changes of nodules.

## Discussion

In human medicine, contrast-enhanced ultrasound (CEUS) has been applied in different clinical settings and guidelines for CEUS study of the spleen has been established in a specific study [21]. Contrast-enhanced ultrasound increases the ability to identify most splenic lesions and represent a reliable diagnostic tool to evaluate lesions as cysts, ectopic splenic tissue, hemangiomas, hamartomas, infarctions, and abscesses, as well as to facilitate the differentiation between benign and malignant lesions. [22–25].

Also in veterinary medicine, studies about the CEUS perfusion pattern of the normal spleen and of focal lesions were performed and most benign splenic lesions showed a perfusion pattern similar to the adjacent parenchyma, so that the lesions were isoechoic to the surrounding normal spleen after the first few seconds and remained homogeneously isoechoic in the wash out phase [12–14]. The authors explained such results by the similar architecture of the vascular network associated with benign hyperplastic conditions and normal spleen [13].

In the present study we applied CEUS by using the contrast agent sulfur hexafluoride microbubbles in order to determine contrast medium behavior in cytological and histological diagnosed focal splenic NLH in 20 dogs.

The appearance of a markedly hypoechoic enhancement to the adjacent spleen 20 to 45 s following the contrast medium inoculation in 95% of examined lesions was the main finding.

In addition, by quantitative analysis, faster TTP and MTT associated to a lesser RBF, and lower PI and RBV in the lesions compared with the surrounding normal parenchyma have been detected, suggesting increased blood velocity and a lower blood volume.

The occurrence of an early wash-in and early wash-out in the NLH observed in our study, does not agree with results of the previous surveys [12–14].

The histologic knowledge about rearrangements of the microvascular environment which can occur in course of nodular lymphoid hyperplasia give us an explanation for the differences on tissue perfusion assessed by Contrast-enhanced ultrasound in NLH lesions and surrounding normal spleen [16].

To justify high perfusion values detected by CEUS analysis in the hyperplastic nodules, Ohlerth et al. (2008) have proposed that distortions of the marginal zone, occurring in the NLH [16], may lead to the failure of the intermediate microcirculation and subsequently to blood stasis within and around the hyperplastic nodule, causing accumulation of microbubbles and thus enhancement of the lesion [14].

Heterogeneous echogenic patterns, characterized by isoechoic regions to the normal spleen, interspersed among anechoic areas, were observed in case of NLH associated with splenic hematomas, and even hematomas without hyperplastic foci were found hypoperfused during CEUS evaluation [13, 29]. However, different perfusion patterns may be seen in hematomas of different ages.

Modifications of the leukocytes environment of the spleen have been seen to affect vascular organization and blood dynamics, nevertheless, hyperplastic lymphoid tissue is known to own a dense vascular bed and to be highly vascularized [14, 18, 20, 26].

This morphological feature do not agree with the isoechoic appearance of NLH to the surrounding parenchyma during all blood pool phases.

In our study, as many as 19 out of a total of 20 focal splenic nodules had early wash-in and wash-out phases than the normal parenchyma. These findings suggest a variation of the blood dynamics in parallel with the reorganization of the parenchyma vascular architecture. The isoechoic feature of the lesions to the normal spleen in the arterial phase prompts us to suppose the lack of alterations of the vascular-type structures responsible for blood supply. By contrast, hypoenhancement of the focal masses at the peak-enhancement and during the wash-out phase of the spleen, and the lower values of RBV and RBF in the lesions compared with the normal surrounding, probably reflect considerably decrease of the vascular spaces, specifically of the venous sinusoids. Other authors have proposed similar vascular arrangements to be responsible of the faster wash-in and wash-out phases in the malignant tumors to the surrounding normal spleen parenchyma and have speculate that the lack of normal sinusoids combined with neoplastic angiogenesis might be one of the causes of a malignant hypoechoic pattern during the late vascular phase [12, 13]. In addition, such a modifications of the intermediate circulation have been observed in splenic lymphoid hyperplasias of dogs infected with visceral leishmaniasis [17, 18, 20].

About the differences during parenchymal phase about hepatic nodular hyperplasia and splenic nodular hyperplasia, Nakamura et al. (2010), although the precise mechanism of splenic parenchymal phase imaging is not fully determined, speculated that the contrast defect during the parenchymal phase created by splenic nodular hyperplasia might be because of a decrease of splenic macrophages [12].

Other factors such as transducer frequency, scanning parameters, contrast agent features, multiple injections of the contrast medium, patient-related factors and operator skills might influence both the subjective assessment and the measurements of perfusion [9, 27–29].

About the contrast agent dose, we used 0.04 ml/kg of body weight, this dosage was lightly different from 0.03 ml/kg previously used in spleen studies with sulphur hexafluoride contrast medium [13, 14, 30–32]. Considering the small volume of contrast medium used for a single bolus/dog, we used 0.04 ml/kg to offset the volume of syringe cone and the difficulty in withdrawing small quantities of contrast agent. This could be considered a limitation of the study. However, too high contrast agent dose results in artefacts such as acoustic shadowing, over-enhancement and signal saturation that are detrimental for quantification of the early phases; too low a dose, with a reduction of the concentration of the microbubbles, afflicting the wash out phases [33]. Concerning the results of the present study, we consider this minimal difference in contrast medium dosage not relevant about the appearance of a markedly hypoechoic enhancement of examined lesions to the adjacent spleen, also considering an equal influence as in NLH lesion as in normal parenchyma.

Another limitation of this study is that the diagnosis of splenic Nodular Lymphoid Hyperplasia was performed in most cases by ultrasound-guided cytologic aspirate and not from histologic samples of the specific lesion. However, also in 48 out of 60 subjects studied by Ohlerth et al. and in benign lesions reported by Rossi et al., the diagnosis derives from ultrasound-guided cytology [13, 14].

Although cytologic diagnoses often reflect histologic results, if missampling or incomplete sampling occurs or tissue architecture is required to distinguish between reactive and neoplastic conditions, accurate diagnosis with fine-needle aspiration may not be possible [34].

However, in a clinical environment and on incidentally detected splenic nodules, although percutaneous fine-needle aspiration and needle core biopsy can be performed safely in dogs with sonographic splenic changes [35], ultrasound-guide fine needle aspirates and following cytology evaluation in addition to an ultrasound follow-up of the lesion are the more common procedure than needle core biopsy or splenectomy.

To date, there are not specific cytologic pattern to determine with high specificity the nature of splenic focal lesion and most of them have been investigated only on histopathology. In fact, cytology, especially if performed as a “quick” and routine diagnostic method, lacks of important features, like, for example, the architecture and distribution of cells as they really appear in the organ or in the lesion. Furthermore, in a routine work, as cytology is often involved, it is quite impossible to discern, with the usual staining methods, the T or B nature of cells, thus, in case of tumor suspect, other techniques must be applied and, therefore, the patient must be otherwise considered. In this order, immunocytochemistry, cytofluorimetry or Polimerase Chain Reaction for antigen receptor rearrangement (PARR) could represent further methods to consider beside the cytology to differentiate a reactive lymphocyte population from a neoplastic one especially when a malignancy is suspected [36].

In this study a scheduled follow up ultrasound of one month after the diagnosis was performed for all dogs; 12/20 subjects have been checked a second time, during a routine clinical examination over the next 6 months. Certainly, a regular control of the lesion for a longer time could have provided greater strength to the citological diagnosis of NLH.

Finally, the CEUS pattern observed in this canine benign splenic lesion was similar to that reported by some authors for splenic malignant neoplastic lesions [7–9]. This finding allowed us to speculate that the specificity of this pattern in canine splenic CEUS study should be reviewed. Additional studies with larger number of patients are needed to determine the clinical utility of contrast-enhanced ultrasound splenic in characterization of splenic nodules.

## Conclusions

The CEUS pattern observed in this canine benign splenic lesion study was similar to that previously reported for splenic malignant neoplastic lesions. Consequently, in clinical practice, attention must be paid to the final diagnosis of canine splenic lesions using only the CEUS exam and only accurate anatomo-vascular studies can clarify the coincidence of similar patterns of the contrast-enhanced ultrasound study in different splenic lesions.

## Methods

### General materials

Informed owners’ consents were obtained. All treatments, housing, and animal care were in compliance with EU Directive 2010/63/EU on the protection of animals used for scientific purposes and with Department’s Animal Ethics Council approval (protocol number: 13/2017).

A CEUS prospective, observational study was performed (October 2015–December 2017) in 20 consecutive dogs with an incidentally detection of a splenic nodular lesion during routine abdominal ultrasonography. Conclusive diagnosis of NLH, after CEUS study, was performed by ultrasound-guided fine needle aspirates (*n* = 16) or histopathologic (*n* = 4) samples taken during splenectomy.

Each dog underwent a physical examination; a red blood cell count and serum biochemistry were performed. In order to avoid adverse reactions due to the microbubble contrast agent, dogs were excluded if, at the physical examination, had evidence of cardiac disease or a history of anaphylactic reactions to vaccines or other medications. Dogs were also excluded if their haematological and haematochemical parameters were not within normal range. An ultrasound follow-up of one month was scheduled for all dogs.

There was one each Yorkshire, Pomeranian, Weimaraner, Hound, West Highland and fifteen mixed breed. Thirteen dogs were male and 7 were female. Their ages ranged from 6 to 13 years (mean 9 years); the bodyweight ranged from 5 to 28 kg (mean 14 kg).

### Ultrasonography procedure

Examinations with B-mode, Doppler ultrasonography and CEUS were performed on all dogs.

B-mode and Doppler ultrasonography of the abdomen were performed by the same operator (C.M.), holding a GPCert (Diagnostic Imaging), during her PhD course and under supervision of a professor of veterinary medical imaging (MDM), using microconvex (5.0 to 8.0-MHz) and linear (10 to 12-MHz) transducers. Spleen tissue was considered normal if the contours were regular and smooth; if the parenchyma was finely textured, homogeneous and more echogenic than liver and the cortex of left kidney. Lesions of the spleen were assessed for their size, number, echo pattern (homogeneous or heterogeneous; hypoechoic, isoechoic, hyperechoic in comparison to normal surrounding parenchyma), and for the presence of cavitary areas within them (anechoic areas surrounded by irregular parenchyma with distal acoustic enhancement or containing corpuscular moveable fluid).

Color Doppler evaluation allowed to evaluate the splenic vascularization, assessing the path of blood vessels in and around the lesions.

CEUS examinations was performed by the same investigator (MDM), using a system equipped with contrast-tuned imaging technology (CnTI Mylab 40 and 60/Vet, Esaote, Genova, Italy) in the not sedated dogs.

After detecting the lesions with standard ultrasound, CEUS was performed with a linear (5.0 MHz) transducer with contrast agent capability; a Mechanical Index (MI) from 0.08 to 0.11, persistency off, a wide dynamic range, and a single focal zone was set deeper to the lesion, including in the image part of normal splenic parenchyma surrounding the lesion.

Eleven exams were done with a machine that allowed to have a double image (B-mode and CnTI images simultaneously); nine exams with a machine with a single image (first fundamental and then CnTI). The contrast agent used was a sulphur hexafluoride signal enhancer (SonoVue®, Bracco International, Milan, Italy), and it was prepared following the manufacturer’s recommendations.

An aliquot (0,04 mL/kg of body weight) of the contrast medium was injected by a second operator (MP), in accordance with a methodology previously reported [37, 38]. Each dog received two bolus injections of contrast agent; the second injection of contrast medium was 10 min later the first. The activation of a timer was performed simultaneously with the contrast agent dose inoculation. Between the two injections, the microbubbles were destroyed with high MI flash on the abdominal aorta. During the first bolus injection, if necessary, machine settings (i.e. total gain, time-gain compensation) were optimized and not changed anymore during the second injection. As animal were not sedated but manually restrained, care was taken to keep the probe in the same position for at least 2 min. Raw data (good-quality video clips for approximately 2 min) obtained during the second contrast enhanced examination were digitally stored on a hard disk and subsequently they were analysed by the same operator.

The splenic focal lesions were classified according to their echogenicity compared to normal tissue in hyperenhancing, isoenhancing, hypoenhancing or not enhancing (anechoic). The presence of homogeneous or heterogeneous lesion patterns, rim enhancement or inner vessels were also evaluated.

Post processing quantitative analysis of video-clips was performed using a dedicated image-analysis software (Qontrast™, Bracco, Milan, Italy) during wash in, peak and wash out phases. For each dog, one Region of Interest (ROI), as large as possible, was manually drawn around the entire lesion. A second one ROI, of a similar size to the previous one, was drawn on the normal parenchyma for comparison. For each ROI, a Time Intensity Curve (TIC) was generated, which is a parametric curve of time (horizontal axis) versus SI, Signal Intensity, (vertical axis). During CEUS the tissue perfusion was evaluated based on video SI variations over time. The maximal SI was defined as a white band in the grey scale bar (8 bit). Within the selected ROI, the software generated the following parameters: Peak Intensity (PI) defined as the percentage increase in SI reached during bolus transit at time T, from baseline intensity to maximal SI; Time to Peak (TTP), defined as the time, in seconds, from T_0_ (injection time) until the maximum SI of the contrast agent. And again, the Regional Blood Flow (RBF) is defined as the ratio between Regional Blood Volume (RBV) and Mean Transit Time (MTT). RBV is proportional to the area under the curve, defined as the area under the time intensity curve during the wash-in and wash-out phase; Mean Transit Time (seconds) was defined as the time interval between half of the maximum SI of the contrast agent in the ascending phase of the curve (wash in) and the same value of SI in the descending phase (wash out).

### Cytology procedure

For each of the 16 cases where FNA was performed, at least three smears were prepared and stained with MGG. In order to assess a cytological judgement and considering the cytology of such lesion overlapping a lymph node cytology, the following criteria were taken into account: cellularity, homogeneous or mixed lymphoid population, more represented cytotype, cytological elimination criteria for neoplasia versus other differential diagnoses [39, 40].

### Statistical analysis

Statistical analysis was performed with a standard computer software program (Graphpad prism, GraphPad Software, Inc., La Jolla, CA 92037 USA). All data were expressed as median and range. To compare perfusion values of normal splenic tissue to the values of the lesion tissue, the Student’s *t*-test was used. When data were non-parametric the Mann-Whitney’s *u-*test was used. To assess a significant association between the type of enhancement of a lesion with the initial diagnosis of benign lymphoid hyperplasia during the blood pool phases, sensitivity with 95% confidence intervals (CI) was calculated. Statistical significance was assessed at the *P* < 0.05 level.

## Data Availability

The datasets used and/or analysed during the current study are available from the corresponding author on reasonable request.

## Abbreviations

CEUS: Contrast-enhanced ultrasonography
CI: Confidence intervals
MGG: May-Grünwald Giemsa
MTT: Mean transit time
NHL: Nodular lymphoid hyperplasia
PI: Peak intensity
RBF: Regional blood flow
RBV: Regional blood volume
ROI: Region of interest
SI: Signal Intensity
TIC: Time-intensity curve
TTP: Time to peak
